# Mean arterial pressure is associated with the neurological function in patients who survived after cardiopulmonary resuscitation: A retrospective cohort study

**DOI:** 10.1002/clc.23441

**Published:** 2020-08-01

**Authors:** Hai‐bo Ai, En‐li Jiang, Ji‐hua Yu, Lin‐bo Xiong, Qi Yang, Qi‐zu Jin, Wen‐yan Gong, Shuai Chen, Hong Zhang

**Affiliations:** ^1^ Rehabilitation Medicine Department The Third Hospital of Mianyang, Sichuan Mental Health Center Mianyang China; ^2^ Rehabilitation Medicine Department The First Affiliated Hospital of Southwest Medical University Luzhou China; ^3^ Rehabilitation Medicine Department Mianyang Central Hospital Mianyang China

**Keywords:** cardiopulmonary resuscitation, mean arterial pressure, neurological function

## Abstract

**Background:**

About 18% to 40% of the survivors have moderate to severe neurological dysfunction. At present, studies on mean arterial pressure (MAP) and neurological function of patients survived after cardiopulmonary resuscitation (CPR) are limited and conflicted.

**Hypothesis:**

The higher the MAP of the patient who survived after CPR, the better the neurological function.

**Method:**

A retrospective cohort study was conducted to detect the relationship between MAP and the neurological function of patients who survived after CPR by univariate analysis, multivariate regression analysis, and subgroup analysis.

**Results:**

From January 2007 to December 2015, a total of 290 cases met the inclusion criteria and were enrolled in this study. The univariate analysis showed that MAP was associated with the neurological function of patients who survived after CPR; its OR value was 1.03 (1.01, 1.04). The multi‐factor regression analysis also showed that MAP was associated with the neurological function of patients survived after CPR in the four models, the adjusted OR value of the four models were 1.021 (1.008, 1.035); 1.028 (1.013, 1.043); 1.027 (1.012, 1.043); and 1.029 (1.014, 1.044), respectively. The subgroups analyses showed that when 65 mm Hg ≤ MAP<100 mm Hg and when patients with targeted temperature management or without extracorporeal membrane oxygenation, with the increase of MAP, the better neurological function of patients survived after CPR.

**Conclusion:**

This study found that the higher MAP, the better the neurological function of patients who survived after CPR. At the same time, the maintenance of MAP at 65 to 100 mm Hg would improve the neurological function of patients who survived after CPR.

## 1 BACKGROUND

Cardiac arrest refers to sudden cardiac arrest accompanied by hemodynamic failure, which leads to severe ischemia, hypoxia, and even death of essential organs (such as the brain).^1^ Although significant progress had achieved on the treatment of cardiac diseases and cardiac arrest, the survival rate of them was still only about 10% to 25%.^2^, ^3^ About 18% to 40% of the survivors of cardiac arrest have moderate to severe neurological dysfunction, preventing a return to work and normal daily activities.^4^ The neurological function of patients with cardiac arrest is related to the sensitivity to ischemic hypoxic brain injury, as well as the duration of cardiac arrest, the quality of cardiopulmonary resuscitation (CPR), the underlying coexisting disease, and post‐resuscitation cerebral tissue perfusion.^5^ Sekhon et alstudy showed that ischemia and hypoxia were very frequent in patients after CPR, and mean arterial pressure (MAP) was firmly related to oxygenation of brain tissue.^6^ At present, studies had shown that the higher the MAP, the less brain damage and the lower the mortality in patients who survived after CPR.^7^, ^8^ At the same time, Jakkula et al study shown that MAP was not associated with the markers of brain tissue damage (neuron‐specific enolase [NSE] and S100B protein).^9^ So, the research about MAP and neurological function of patients survived after CPR is limited and conflicted. In this study, we hope to furtherly explore the relationship between MAP and the neurological function of patients who survived after CPR.

## 2 METHODS

### 2.1 Study design

A Retrospective cohort study.

### 2.2 Objective

To explore the relationship between MAP and the neurological outcome of patients who survived after CPR.

### 2.3 Data source

The data in this study were provided by Fabio Silvio Taccone and stored in Dryad Database (https://datadryad.org/resource/doi:10.5061/dryad.qv6fp83).^10^, ^11^

### 2.4 Evaluation of neurological function

The neurological function was evaluated at 3 months, according to the cervical performance categories score (CPC), favorable neurological outcome (CPC: 1‐2), and unfavorable neurological outcome (CPC: 3‐5).

### 2.5 Inclusion criteria

Patients who survived after CPR and admission to intensive care unit (ICU).

### 2.6 Exclusion criteria

(a) Patients with previous neurological impairment and (b) Patients with the missing value of MAP or 3‐month neurological function score.

### 2.7 Participants

From January 2007 to December 2015, a total of 347 patients survived after CPR and admitted to ICU. Fifty‐seven patients were excluded, including 52 patients with previous neurological impairment, four cases with MAP deletion, and once case with 3‐month neurological function score deletion. Finally, 290 patients met the inclusion and exclusion criteria.

### 2.8 Post‐resuscitation care

The protocol of post‐resuscitation care has been extensively described elsewhere, widely accepted, and applied.^12^, ^13^, ^14^ All coma patients with cardiac arrest received targeted temperature management (TTM, target body temperature: 32°C‐34°C for 24 hours, passive rewarming <0.5 C/h). Midazolam, morphine, and cisatracurium were used for deep sedation to control shivering. Pulse index continuous cardiac output was used for hemodynamic monitoring. Repeated transesophageal and transthoracic echocardiography were applied to evaluate cardiac function. The MAP was maintained at 65 to 70 mm Hg by volume resuscitation, dobutamine, or norepinephrine. Extracorporeal membrane oxygenation (ECMO) would be involved in patients with severe cardiogenic shock. Arterial oxygen saturation was maintained above 94% and normocapnia by mechanical ventilation. Blood glucose was kept at 110 to 150 mg/dL by continuous insulin infusion. Enteral nutrition initiated during TTM and then continued according to stomach tolerance.

### 2.9 Clinical and biochemical data collection

(a) Demographic characteristics: Age, sex, and weight; (b) Existing chronic diseases: chronic heart failure, hypertension, coronary heart disease, diabetes, chronic obstructive pulmonary disease (COPD), chronic renal failure, and liver cirrhosis; (c) Detailed information of CPR: out‐of‐hospital or hospital CPR, witness arrest, bystander CPR, initial rhythm, etiology of CPR, time to return of spontaneous circulation (ROSC), total adrenaline dose, corticoids; (d) Laboratory detection indexes: lactate dehydrogenase (LDH), activated partial thromboplastin time (APTT), blood glucose (Glu), PH, arterial partial pressure of carbon dioxide (PaCO_2_), arterial partial pressure of oxygen (PaO_2_), MAP, lactate (Lac), CRP, creatinine; (e) Disease severity score: sequential organ failure assessment (SOFA), and acute physiological and chronic health assessment II score (APACHE II score); (f) Organ function support therapies, including mechanical ventilation, Target temperature management (TTM), extracorporeal membrane oxygenation (ECMO), and continuous renal replacement therapy, were collected during ICU.

### 2.10 Statistical methods

(a) Statistical description: Mean ± SD (x ± s) was used for continuous variables and counts variables were shown by numerical values and percentages. (b) Univariate analysis and multi‐factor regression analysis were used to detect the relationship between MAP and the neurological function of patients who survived after CPR. (c) Selection of adjusted variables for multi‐factor regression analysis: Since only 130 samples with good neurological function, to avoid over‐fitting of the model, we controlled the number of the adjusted variables less than 13. At the same time, to make the model results more reliable, we adopted two ways to select the adjusted variables for multi‐factor regression analysis, respectively: (a) According to the clinical significance and *P*‐value <.1 (SOFA score and APACHE II score included MAP, so SOFA and APACHE II score were not included in multi‐factor regression analysis to avoid collinearity). Finally, age, diabetes, COPD, out of hospital cardiac arrest, non‐cardiac epidemiology, non‐shockable rhythm, bystander CPR, witnessed arrest, total epinephrine dose, time to ROSC, acute kidney injury (AKI), lac, and TTM were adjusted in multi‐factor regression analysis. (b) If a variable was added to the model, which caused the change of the effective value to be more than 10%, the variable would be adjusted in multi‐factor regression analysis. Finally, bystander CPR, total epinephrine dose, and non‐shockable rhythm were selected for multi‐factor regression analysis. Subgroups analysis were performed, according to MAP, shock, hypertension, SOFA score, ECMO, and TTM, respectively. Only bystander CPR, total epinephrine dose, and non‐shockable rhythm were adjusted to avoid over‐fitting of the model in subgroup analysis.

## 3 RESULTS

### 3.1 The clinical characteristics of patients

A total of 290 patients met the inclusion and exclusion criteria and were included in this study, among which 130 cases (44.83%) were favorable neurological outcome at 3 months. The mean age was 61.35 ± 15.65 years, the male/female ratio was 79/211, the coronary artery disease was 120 (41.38%), diabetes was 70 (24.14%), chronic failure was 47 (16.26%), COPD was 49 (16.90%), chronic heart failure was 66 (22.76%), hypertension was 117 (40.34%), non‐cardiac etiology was 102 (35.17%), non‐shockable rhythm was158 (54.48%), out‐of‐hospital cardiac arrest was 163 (56.21%), time to ROSC was 18.81 ± 14.75 minutes, total epinephrine dose was 4.23 ± 3.82 mg, and MAP was 90.68 ± 20.82 mm Hg (Table 1).

**TABLE 1:** The clinical characteristics of patients

Variables	Mean ± SD, N (%)
Age (y)	61.35 ± 15.65
Sex(F/M)	79/211
Weight (kg)	77.12 ± 14.78
Coronary artery disease, n (%)	120 (41.38%)
Diabetes, n (%)	70 (24.14%)
Chronic renal failure, n (%)	47 (16.26%)
COPD, n (%)	49 (16.90%)
Chronic heart failure, n (%)	66 (22.76%)
Hypertension, n (%)	117 (40.34%)
Witnessed arrest, n (%)	248 (85.52%)
Bystander CPR, n (%)	193 (66.55%)
Out of hospital cardiac arrest, n (%)	163 (56.21%)
Non‐cardiac etiology, n (%)	102 (35.17%)
Non‐shockable rhythm, n (%)	158 (54.48%)
Time to ROSC (min)	18.81 ± 14.75
Epinephrine total dose (mg)	4.23 ± 3.82
MAP (mm Hg)	90.68 ± 20.82
AKI, n (%)	164 (56.55%)
APACHE II score	24.13 ± 7.10
SOFA score	10.72 ± 3.44
Glu (mg/dL)	237.78 ± 116.64
pH	7.29 ± 0.13
PO2 (mm Hg)	155.04 ± 109.09
PCO2 (mm Hg)	38.96 ± 9.56
APTT (sec)	43.44 ± 29.55
PT (sec)	63.48 ± 22.53
Lac (mEq l^−1^)	6.11 ± 3.09
TTM, n (%)	258 (89.27%)
Mechanical ventilation, n (%)	286 (98.62%)
CRRT, n (%)	39 (13.45%)
ECMO, n (%)	36 (12.41%)
Shock, n (%)	152 (52.41%)
Favorable neurological outcome at 3 mo, n (%)	130 (44.83%)

Abbreviations: AKI, acute kidney injury; APACHE II score, acute physiological and chronic health assessment II score; COPD, chronic obstructive pulmonary disease; CPR, cardiopulmonary resuscitation; ECMO, extracorporeal membrane oxygenation; CRRT, continuous renal replacement therapy; Lac, lactate; MAP, mean arterial pressure; ROSC, return of spontaneous circulation; SOFA, sequential organ failure assessment; TTM, targeted temperature management.

### 3.2 The results of univariate analysis

Univariate analysis showed that age, non‐cardiac epidemiology, non‐shockable rhythm, witnessed arrest, bystander CPR, time to ROSC (min), total epinephrine dose, SOFA, MAP, AKI, and shock were associated with the neurological outcome of patients who survived after CPR (*P* < .05).(see Table 2).

**TABLE 2:** Univariate analysis

Variables	Statistics	Favorable neurological outcome at 3 mo
Age	61.35 ± 15.65	0.98 (0.97, 1.00), 0.023
Sex	79/211	1.10 (0.66, 1.86), 0.708
Weight	77.12 ± 14.78	1.01 (0.99, 1.03), 0.258
Chronic heart failure	66 (22.76%)	0.75 (0.43, 1.31), 0.313
Hypertension	117 (40.34%)	1.23 (0.77, 1.97), 0.393
Coronary artery disease	120 (41.38%)	0.90 (0.56, 1.44), 0.667
Diabetes	70 (24.14%)	0.61 (0.35, 1.06), 0.080
COPD	49 (16.90%)	0.54 (0.28, 1.03), 0.063
Chronic renal failure	47 (16.26%)	0.72 (0.38, 1.37), 0.315
Out of hospital	163 (56.21%)	1.12 (0.70, 1.78), 0.646
Non‐cardiac etiology	102 (35.17%)	0.51 (0.31, 0.84), 0.008
Non‐shockable rhythm	158 (54.48%)	0.27 (0.16, 0.43), <0.001
Chronic anticoagulation	53 (18.28%)	0.93 (0.51, 1.70), 0.817
Witnessed arrest	248 (85.52%)	2.27 (1.11, 4.64), 0.025
Bystander CPR	193 (66.55%)	1.96 (1.18, 3.25), 0.009
Time to ROSC (min)	18.81 ± 14.75	0.97 (0.95, 0.99), <0.001
Epinephrine total dose (mg)	4.23 ± 3.82	0.84 (0.77, 0.91), <0.001
TTM	258 (89.27%)	0.55 (0.26, 1.18), 0.125
APACHE II	24.13 ± 7.10	0.97 (0.94, 1.00), 0.052
SOFA	10.72 ± 3.44	0.87 (0.81, 0.93), <0.001
Lac	6.11 ± 3.09	0.93 (0.86, 1.01), 0.075
APTT	43.44 ± 29.55	1.00 (0.99, 1.01), 0.515
Glu	237.78 ± 116.64	1.00 (1.00, 1.00), 0.503
pH	7.29 ± 0.13	1.83 (0.31, 10.82), 0.506
PO2	155.04 ± 109.09	1.00 (1.00, 1.00), 0.771
PCO2	38.96 ± 9.56	1.00 (0.98, 1.03), 0.903
MAP	90.68 ± 20.82	1.03 (1.01, 1.04), <0.001
PT	63.48 ± 22.53	1.01 (1.00, 1.02), 0.117
Mechanical ventilation	286 (98.62%)	0.00 (0.00, Inf), 0.983
CRRT	39 (13.45%)	0.94 (0.48, 1.86), 0.867
AKI	164 (56.55%)	0.46 (0.29, 0.74), 0.001
IABP	20 (6.90%)	0.50 (0.19, 1.35), 0.174
ECMO	36 (12.41%)	0.98 (0.49, 1.98), 0.961
Shock	152 (52.41%)	0.50 (0.32, 0.81), 0.004

Abbreviations: AKI, acute kidney injury; APACHE II score, acute physiological and chronic health assessment II score; COPD, chronic obstructive pulmonary disease; CPR, cardiopulmonary resuscitation; CRRT, continuous renal replacement therapy; ECMO, extracorporeal membrane oxygenation; Lac, lactate; MAP, mean arterial pressure; ROSC, return of spontaneous circulation; SOFA, sequential organ failure assessment; TTM, targeted temperature management.

### 3.3 The results of multi‐factor regression analysis

Multi‐factor regression analysis showed that MAP was associated with the neurological outcome of patients who survived after CPR. The adjusted OR of model 1 was 1.021 (1.008, 1.035) after adjusting for age, diabetes, COPD, out of hospital cardiac arrest, non‐cardiac etiology, and non‐shockable rhythm. The adjusted OR of model 2 was 1.028 (1.013, 1.043) after adjusting age, diabetes, COPD, out of hospital cardiac arrest, non‐cardiac epidemiology, non‐shockable rhythm, bystander CPR, witnessed arrest, total epinephrine dose, and time to ROSC. The adjusted OR of model 3 was 1.027 (1.012, 1.043) after adjusted for age, diabetes, COPD, out of hospital cardiac arrest, non‐cardiac epidemiology, non‐shockable rhythm, bystander CPR, witnessed arrest, total epinephrine dose, time to ROSC, AKI, Lac, and TTM. The adjusted OR of model 4 was 1.029 (1.014, 1.044) after adjusting bystander CPR, total epinephrine dose, and non‐shockable rhythm (Table 3).

**TABLE 3:** Multivariate logistic regression that investigated the relationship between MAP and the favorable neurological outcome at 3 months in patients with successful CPR in ICU

Model	Non‐adjusted OR (95% CI), *P*‐Value	Adjust OR (95% CI), *P*‐Value
MAP		
Model 1	1.025 (1.013, 1.038), <.001	1.021 (1.008, 1.035), .002
Model 2	1.025 (1.013, 1.038), <.001	1.028 (1.013, 1.043), <.001
Model 3	1.025 (1.013, 1.038), <.001	1.027 (1.012, 1.043), <.001
Model 4	1.025 (1.013, 1.038), <.001	1.029 (1.014, 1.044), <.001

*Note:* Model 1: Adjusted for age, diabetes, COPD, out of hospital cardiac arrest, non‐cardiac etiology, non‐shockable rhythm.

*Note:* Model 2: Adjusted for model 1 and bystander CPR, witnessed arrest, epinephrine total dose, time to ROSC.

*Note:* Model 3: Adjusted for model 2 and AKI, Lac, TTM.

*Note:* Model 4: Adjusted for bystander CPR, epinephrine total dose, non‐shockable rhythm.

Abbreviations: AKI, acute kidney injury; COPD, chronic obstructive pulmonary disease; CPR, cardiopulmonary resuscitation; Lac, lactate; MAP, mean arterial pressure; ROSC, return of spontaneous circulation; TTM, targeted temperature management.

### 3.4 The results of subgroup analysis of multi‐factor regression analysis

Subgroup analysis found that when 65 mm Hg ≤ MAP<100 mm Hg, with the increase of MAP, the neurological outcome of patients who survived after CPR also increased. However, when MAP<65 mm Hg or 100 mm Hg ≤ MAP, with the rise of MAP, the neurological outcome of patients who survived did not improve. Subgroup analysis also found that when patients with TTM or without ECMO, with the increase of MAP, the neurological outcome of patients who survived also improve. Finally, we also found that with the rise of MAP, the neurological outcome of patients who survived after CPR would be enhanced, regardless of whether the patient with hypertension or with the SOFA score ≥ 12 or < 12 (Table 4).

**TABLE 4:** Subgroup analysis of multivariate logistic regression that investigated the relationship between MAP and the favorable neurological outcome at 3 months in patients with successful CPR in ICU

Subgroup variables	Non‐adjusted OR (95% CI), *P*‐Value	Adjust OR (95%CI), *P*‐Value
MAP		
MAP <65 mm Hg	0.766 (0.583, 1.006), .055	0.704 (0.473, 1.047), .083
65 mm Hg ≤ MAP<100 mm Hg	1.044 (1.012, 1.078), .008	1.043 (1.005, 1.082), .026
100 mm Hg ≤ MAP	1.014 (0.982, 1.047), .388	1.020 (0.984, 1.057), .274
Shock		
Yes	1.030 (1.010, 1.050), .003	1.035 (1.013, 1.059), .002
NO	1.017 (1.001, 1.034), .037	1.025 (1.005, 1.045), .014
Hypertension		
Yes	1.018 (1.000, 1.036), .051	1.027 (1.005, 1.049) .014
No	1.032 (1.014, 1.049), <.001	1.032 (1.012, 1.052), .002
SOFA score		
≥12	1.032 (1.008, 1.056), .009	1.032 (1.003, 1.063), .032
< 12	1.023 (1.008, 1.038), .003	1.025 (1.007, 1.042), .005
ECMO		
Yes	1.029 (0.989, 1.070), .164	1.031 (0.978, 1.087), .251
No	1.025 (1.012, 1.038), <.001	1.029 (1.014, 1.045), <.001
TTM		
Yes	1.026 (1.013, 1.040), <.001	1.030 (1.015, 1.045), <.001
No	1.020 (0.969, 1.074), .441	1.015 (0.955, 1.079), .623

*Note:* Adjusted variables: bystander CPR, epinephrine total dose (mg), non‐shockable rhythm.

Abbreviations: AKI, acute kidney injury; COPD, chronic obstructive pulmonary disease; CPR, cardiopulmonary resuscitation; ECMO, extracorporeal membrane oxygenation; Lac, lactate; MAP, mean arterial pressure; ROSC, return of spontaneous circulation; SOFA, sequential organ failure assessment; TTM, targeted temperature management.

## 4 DISCUSSION

No matter univariate analysis, multi‐factor regression analysis, or multivariate subgroup analysis, all showed that MAP was associated with the neurological outcome of patients who survived after CPR, with the increase of MAP, the neurological outcome of patients also improved.

Brain tissue is susceptible to ischemia and hypoxia. When cardiac arrest occurs, consciousness disorder may occur within 5 to 10 seconds, pupil dilation may occur within 30 to 60 seconds, and irreversible brain injury may occur after cardiac arrest exceeds 4 to 6 minutes.^15^ Cerebral blood flow depends on cerebral perfusion pressure, which is equal to MAP minus intracranial pressure. Therefore, MAP is one of the essential factors affecting cerebral blood flow.^16^ Govindan et al measured the hemoglobin in brain tissue by using the external spectrum technique, and found that the circulating hemoglobin in brain tissue was correlated with MAP, and also positively correlated with brain tissue damage.^17^ According to Hirose et al study, when the MAP dropped during the cesarean section, the regional cerebral blood volume and oxygenation also dramatically declined.^18^

This study found that the higher the MAP, the better the neurological outcome of patients who survived after CPR. The possible reasons were as follows: (a). the cerebral vascular autonomic regulation function would be lost due to ischemia and hypoxia of brain tissue after CPR.^19^ (b) Cessation of blood flow during a cardiac arrest could result in microvascular thrombosis,^13^ the formation of micro thrombosis would lead to an increase of vascular resistance and an increasing dependence of cerebral blood flow on the MAP.

This study also found that when 65 mm Hg ≤ MAP<100 mm Hg, with the increase of MAP, the neurological function of patients who survived after CPR was better. However, when MAP<65 mm Hg or 100 mm Hg ≤ MAP, with the rise of MAP, the neurological function did not increase. The possible reasons were as follows: (a) when MAP<65 mm Hg, it would lead to hypoperfusion of brain tissue. Hypoperfusion would further lead to ischemia and hypoxia of brain tissue and increase cerebral edema, which would also lead to hypoperfusion of brain tissue, and eventually lead to the aggravation of brain injury and the long‐term deterioration of nerve function.^20^ (b) When 100 mm Hg ≤ MAP, it would lead to congestive reperfusion, aggravating cerebral edema, and reperfusion injury, due to impaired cerebrovascular autonomic regulation after cardiac arrest.^20^, ^21^, ^22^

According to the 2019 AHA guidelines for cardiopulmonary resuscitation, there was no sufficient evidence to recommend ECMO for routine use. Only when conventional CPR efforts failed, and skilled physicians could quickly establish ECMO, ECMO might be considered as a rescue treatment.^23^, ^24^ Our study found that ECMO did not improve the neurological function of patients who survived after CPR, which might be due to the delay of ECMO. Because all the patients included in this study, only in the case of inadequate response to conventional treatment, ECMO would be considered as a last resort. At this time, patients had complicated with multiple organ failures, which leads to the fact that ECMO could not improve the neurological function of patients who survived after CPR.

Studies have shown that TTM could improve the survival and neurological function of patients who survived after CPR.^25^ Our research found that the higher MAP of patients treated with TTM, the better the neurological function of patients who survived after CPR. However, for non‐TTM patients, MAP was not associated with the neurological function of patients who survived after CPR. The possible reason was that patients treated with TTM were with coma, while non‐TTM patients were awake. At the same time, Grand et al research also found that in survivors with impaired cognitive function, MAP during TTM was significantly higher.^26^

Our study also found that whether patients complicated with or without shock, hypertension, and SOFA scores ≥12, with the increase of MAP, the better the neurological function of patients who survived after CPR. It further confirmed that the MAP was associated with the neurological outcome of patients who survived after CPR.

At present, there had several studies detected the relationship between MAP and prognosis of patients with cardiac arrest. Jakkula et al study included 120 outside the hospital cardiac arrest patients, found that MAP was associated with NSE and S100B protein (NSE and S100B were often used to evaluate the degree of brain injury).^27^

Ameloot et al study included 112 out‐of‐hospital cardiac arrest patients, found that higher than reduces brain ischemia, and improves outcome.^8^ John Bro‐Jeppesen et al study showed that a low MAP was associated with increased mortality.^7^ As compared with these studies, our study had the following characteristics: (a). Our study not only included patients with out‐of‐hospital cardiac arrest, but also patients with in‐hospital cardiac arrest, so the findings of our research might be more adaptive. (b) The sample size of our study was relatively more massive, so the credibility of our conclusions might be more substantial. (c) In our study, the variables that might affect the prognosis of cardiac arrest were adjusted to make our findings more reliable. (d) The above studies mainly focused on the relationship between MAP and the short‐term outcome of CPR. At the same time, there were relatively few studies on the neurological outcome of cardiac arrest after CPR. (e) Our study provided a new theoretical basis for controlling the MAP after CPR from 65 to 100 mm Hg. Previous guidelines suggested that the MAP after CPR should be controlled between 65 and 100 mm Hg according to animal experiments or other studies on nerve injury.

### 4.1 Limitations of the study

Our study was a retrospective study, so a prospective study should be conducted to verify the findings of our research further. The MAP of our study obtained at ICU admission, it is necessary to investigate the relationship between MAP dynamics and the neurological function of patients who survived after CPR, which would be with more clinical value. Since our study belongs to the reuse of data, the myocardial infarction, emergent catheterization, cardiac etiology, and all‐death in our study were missing in the original data, which may be with potential confounders.

## 5 CONCLUSION

This study found that the higher MAP, the better the neurological function of patients who survived after CPR. At the same time, the maintenance of MAP at 65 to 100 mm Hg would improve the neurological function of patients who survived after CPR.

## CONFLICT OF INTEREST

The authors declare no potential conflicts of interest.

## AUTHORS CONTRIBUTIONS

Hai‐bo Ai, En‐li Jiang, and Ji‐hua Yu wrote the manuscript; Lin‐bo Xiong, Qi Yang, and Qi‐zu Jin finished the statistical analysis; Wen‐yan Gong and Shuai Chen were responsible for checking and correction, Hong Zhang was responsible for research design and process guidance.

## ETHICS STATEMENT

New ethics approval was not applicable, because the original author had obtained the ethical approval when conducting this study. Permission to participate was also not appropriate, because our review was a retrospective study of data reuse, and the message of the patients was anonymous.

## Supporting information


**Supplementary figure 1** ROC analysis of MAP for the prediction of neurological function
