# Supplementary figures and images for: Mean arterial pressure is associated with the neurological function in patients who survived after cardiopulmonary resuscitation: A retrospective cohort study
Source: Clin Cardiol. 2020 Aug 1;43(11):1286–93. doi: 10.1002/clc.23441 (PMC7661647; doi:10.1002/clc.23441)

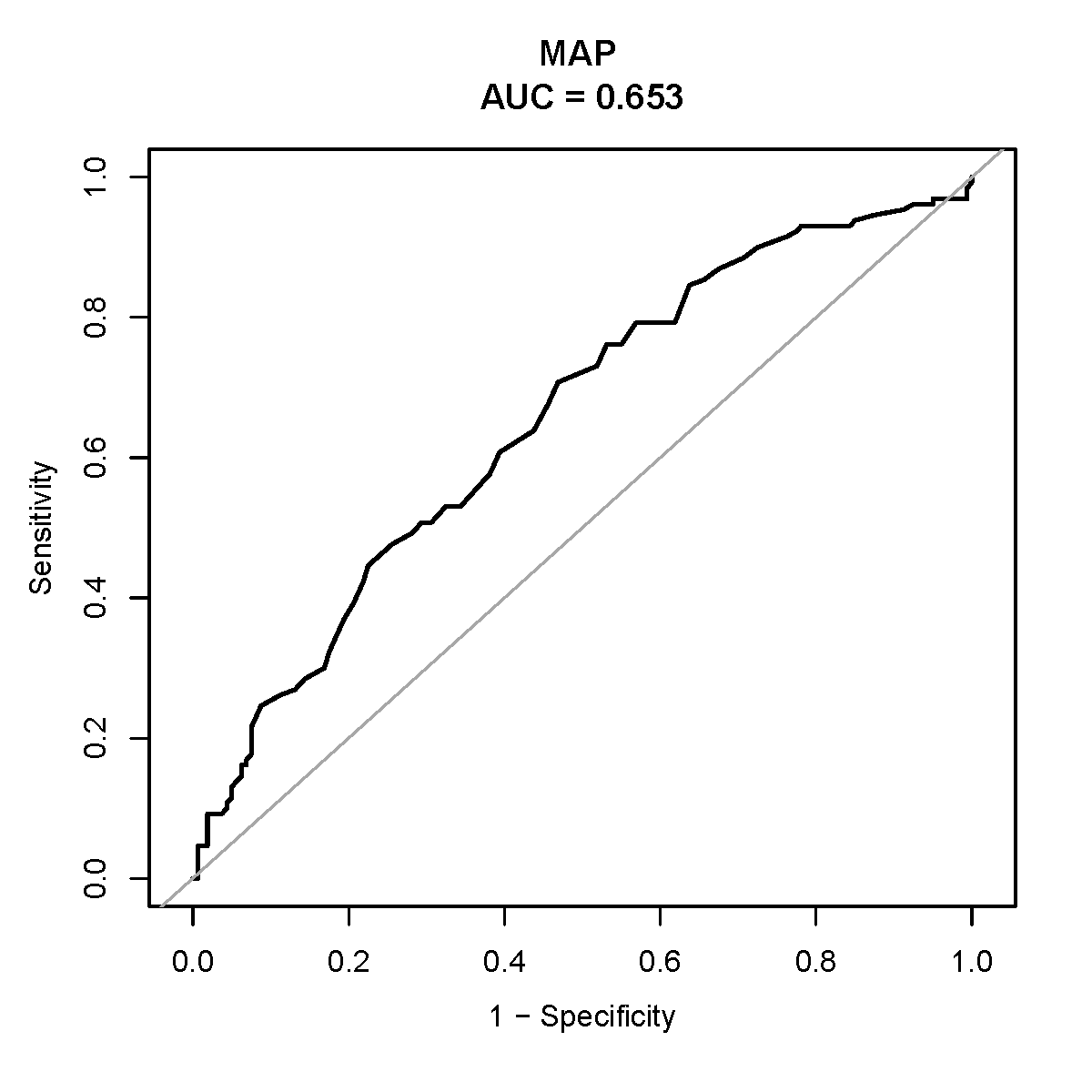

Supplement: Supplementary file 1 — Supplementary figure 1 ROC analysis of MAP for the prediction of neurological function [file CLC-43-1286-s001.tiff]
